# Assessment of the availability of sexual and reproductive healthcare for Venezuelan migrant women during the SARS-CoV-2 pandemic at the north-western border of Brazil-Venezuela

**DOI:** 10.1016/j.jmh.2022.100092

**Published:** 2022-03-17

**Authors:** Luis Bahamondes, Maria Y Makuch, Deborah Margatho, Charles M. Charles, Cinthia Brasil, Helder SF de Amorin

**Affiliations:** a Department of Obstetrics and Gynaecology, Faculty of Medical Sciences, University of Campinas (UNICAMP), Caixa Postal 6181, Campinas, SP 13084-971, Brazil; b Direction of Basic Attention Care, Health Secretary, Municipality of Boa Vista, Boa Vista, RR, Brazil; c Direction of Primary Healthcare, Health Secretary, State of Roraima, Boa Vista, RR, Brazil

**Keywords:** Migrants, Venezuela, Brazil, Sexual and reproductive health, Covid-19

## Abstract

**Background:**

The public healthcare system of Roraima state, at the north-western border Brazil-Venezuela, experienced an impact due to the incoming of Venezuelan migrants and to the SARS-CoV-2 (COVID-19) pandemic. After the beginning of the pandemic, the Brazilian government closed the border; however, migration continued through informal paths. There is scarce information regarding the availability of sexual and reproductive health (SRH) services at settings impacted both by migration and the COVID-19 pandemic. Consequently, we aimed to assess the availability and access to SRH services for Venezuelan migrant at Roraima state during the COVID-19 pandemic.

**Methods:**

We conducted a cross-sectional study with data collected during the pandemic using an electronic questionnaire. We interviewed three policy makers responsible for the directorate of primary care both at the municipal and state level; the directors of the two public hospitals and the two referral centres for women's health in Boa Vista, and in Pacaraima the director of the hospital and of the basic health post. We also interviewed 20 out of the 34 (58.8%) managers of the basic health posts in Boa Vista and 10 healthcare providers (physicians and nurses). Data collected covered issues pertaining to access to SRH services, and explored the impacts of the COVID-19 on continuation, access, and use of SRH services.

**Results:**

It was reported that 60% of the beds in the public sector were reallocated for COVID-19 cases and 26 out of the 34 (76%) health posts assessed interrupted the provision of SRH services, including contraceptive provision and gynaecological consultation, since these were considered not essential services. Most of the participants, (25/36; 69.4%) reported that both the state and the municipal health authorities implemented community outreach using telephone, social media, radio, and television, with guidance for the population on how to seek care using the healthcare network.

**Conclusions:**

The healthcare system in Roraima was impacted firstly by the need to provide SRH to a large number of Venezuelan migrant women, and after an effort to adapt to the reality that this migrant crisis posed; this system was affected by the needs to adapt to the COVID-19 pandemic.

## Highlights

• Access to essential SRH services.
• Less SRH services offered.
• Lack of availability and access to contraceptives.
• Impact on health infrastructure.
• Reallocation of beds to attend COVID-19 cases.
• Shortage of healthcare providers.
• Challenges and opportunities for strengthening SRH services.
• Doubts about the management of the pandemic due to conflicting messages.

## 1 Introduction

It is estimated that the number of migrants and refugee is 71 million people worldwide and half of them are women (Askew et al., 2016; UNHCR, 2020; World Migration Report, 2020). The migration process affects the lives and health displaced persons and increase inequalities mainly to access to healthcare (Hussein, 2020). Since 2015, in Venezuela has been undergoing an economic and social situation (Reuters, 2018; UNHCR, 2019a; UNHCR, 2019b) that has led to a crisis in the national health system with deleterious effects on many health indicators, including sexual and reproductive health (SRH) (WHO, 2012; CDC, 2021). Of the 5.6 million Venezuelans who emigrated to other Latin American countries, it has been estimated that 300,000 entered Brazil through Roraima, the main migration entry point, one of the smallest Brazilian states located at the north-western border with Venezuela (UNHCR, 2020a; 2020b; World Migration Report, 2020). In a humanitarian effort the United Nations High Commissioner for Refugees (UNHCR) in collaboration with the Brazilian federal government and the Brazilian army has established 15 shelters in Roraima to receive these migrants before resettlement to other regions of the country is possible (UNHCR, 2020a; 2020b; World Migration Report, 2020).

The constant incoming number of migrants posed challenges and strains for the local public healthcare system, with a consequent impact on SRH care. In Brazil, all public healthcare is provided by the National Health System (NHS) (*Sistema Unico de Saúde, SUS*), at no cost to nationals, legal residents, migrants, and non-legally documented people (Campos, 2018; Kleinert and Horton, 2011). Nevertheless, in previous quantitative and qualitative reports from our group based on data collected before the pandemic we identified that the interviewed Venezuelan women at reproductive age reported important unmet needs in the attention they received in SRH including the difficulties to access to contraceptive methods, particularly long-acting reversible contraceptives (LARC), almost one quarter of pregnant women failed to receive any antenatal (ANC) or postnatal care (Bahamondes et al., 2020; Makuch et al., 2021).

In Brazil, the first case of SARS-CoV-2 (COVID-19) was detected on February 26, 2020 and the first COVID-19-related death was reported on March 17, 2020 being up today 624,000 related deaths (Brasil, 2021). Since then a disruption and challenges have occurred in healthcare services (Haldane et al., 2021), such as shortages in healthcare professionals, and limited resources, including testing capacity, equipment, intensive care unit beds, and medicines and women were also confronted with difficulties to access to SRH services (Hussein, 2020).

Due to the pandemic, the border Brazil-Venezuela was closed on March 18, 2020 (Brasil 2020a, Brasil 2020b) and in the following months the UNHCR closed four of the 15 shelters established in Roraima. However, migrants continued arriving in this region and at the beginning of 2021, 1603 non-legally documented Venezuelans were living outside the UN shelters and on the streets or in very precarious settlements (IOM, 2021; Soeiro et al., 2021).

Due to the scarce information on the availability of SRH services within a complicated migration situation, the growing demand for healthcare services, the adaptation of the services for this demand, and the strains pose by the COVID-19 pandemic; our objectives were to assess the availability of and access to essential SRH services, including the impact on perinatal and maternal health, amongst Venezuelan migrant women, at the north-western border of Brazil-Venezuela during the COVID-19 pandemic.

## 2 Materials and methods

### 2.1 Study design

We conducted a cross-sectional study between June 2020 and July 2021 to assess availability of and access to essential SRH services. We followed the World Health Organisation (WHO) definition of SRH which includes pregnancy, fertility, cervical screening, contraception, and sexually transmitted infections (WHO, 2020). The study was approved by the *Comité de Ética em Pesquisa, Universidade Estadual de Campinas*, Campinas, SP, Brazil, (#3,620,337; #20,458,219.0.0000.5404). All participants signed a written or electronic informed consent form.

### 2.2 Setting

The study was conducted at the main gate of Venezuelan migrants to Brazil through the north-western border between the counties. Roraima is one of the smallest and poorest Brazilian states, with a population of 631,000 inhabitants. The study was conducted at two sites of the state: Boa Vista and Pacaraima. Boa Vista, the state capital has 419,000 inhabitants, has two public hospitals, one devoted to obstetrics and gynaecology and the other one a general hospital, two referral centres for women's health, and a primary healthcare network with 34 basic health posts and 61 teams to care for family health (Soeiro et al., 2021); one central laboratory for routine exams and agreements with private laboratories to perform the minimal package of analysis established by the Ministry of Health for ANC and more complex analyses. Pacaraima, a small city 200 km from Boa Vista, has 17,000 inhabitants, one small hospital and one basic health post.

Before the migration crisis, the public maternity hospital already provided attention to pregnant Venezuelan women. During 2017, 5.2% of the deliveries were of Venezuelan women, which increased to 26.1% in 2019 and declined to 21.6% in 2020 after the border was closed. In 2014, 179 Venezuelan women gave birth, in 2019 3170 in 2019 and in 2020 2300 (Barreto et al., 2018; Brasil 2021b, 2021c, 2021d). At that time, the two Municipal Health Secretariats (Boa Vista and Pacaraima cities), the State Health Secretariat of Roraima, and the international humanitarian agencies had organised strategies to provide essential healthcare including SRH for Venezuelan migrants (Bahamondes et al., 2020; Makuch et al., 2021; FGV, 2020).

### 2.3 Data tools and collection

Data collection was conducted using an electronic questionnaire that specifically assessed the impact of the COVID-19 pandemic on health services, adapted from a tool developed by the UNFPA and Save the Children and translated into Portuguese (UNFPA and Save the Children, 2009; IAWG, 2017). The survey covered the position of the respondents and the characteristics of the facilities: number of beds and how many were re-allocated to COVID-19 cases, problems and the impact on SRH services, implementation of digital technology or telehealth services to provide consultations and messages about SRH issues within the pandemic context, provision of an essential package of SRH, and problems that emerged at the time of the pandemic. Interviewees also responded to questions regarding data on the number of medical consultations during the three months before the interviews, obtained from the statistics registered at the different facilities. In addition, we obtained some epidemiological data regarding perinatal and maternal health, number of births and maternal and child mortality from the Roraima State Epidemiological Surveillance Department. The questionnaire was sent and completed electronically via Google forms (Google LLC, Mountain View, CA, USA) due to the restrictions pose to the pandemic to conduct face-to-face interviews.

We interviewed the three policy makers responsible for the directorate of primary care both at the municipal and state level; the directors of the two public hospitals and the two referral centres for women's health in Boa Vista, and in Pacaraima the director of the hospital and of the basic health post. We also interviewed 20 out of the 34 (58.8%) managers of the basic health posts in Boa Vista which were located near to the 13 shelters established by the UNHCR and seven health posts which attended, according to the information provided by the Health Municipal Secretariat, a large number of migrants. We also interviewed 10 healthcare providers (physicians and nurses) working at the health posts selected based on their availability of time and willingness to participate. Data was collected on the roles and responsibilities of the respondents, availability of services at outpatient clinics and at hospitals regarding ANC, childbirth and postnatal care and eventual complications, access to contraceptives, availability of services for gender-based violence (GBV) and materials for detection and treatment of HIV and other sexual transmitted infections (Box 1).

**Box 1:** Main themes on SRH care services, maternal and perinatal health indicators and accessibility alternatives evaluated.

***Impact on SRH services and infrastructure***
• Provision of an essential package of SRH
• Number of SRH consultations during the three months before the interviews
• Availability of services at outpatient clinics and at hospitals regarding ANC
• Number of beds re-allocated to COVID-19
• Childbirth and postnatal care and eventual complications
• Access to contraceptive methods
• Availability of services for GBV
• Availability and access to services related to HIV and other STIs
***Accessibility***
• Implementation of digital technology or telehealth services to provide consultations
• Messages about SRH issues within the pandemic context
***Impact on perinatal and maternal health***
• Perinatal and maternal health
• Number of births
• Number of maternal and child death
***Challenges and opportunities for strengthening SRH services***

ANC: antenatal care; SRH: Sexual and reproductive health; GBV: gender-based violence; STIs sexually transmitted infections.

### 2.4 Data analysis

The information collected through the electronic interviews and the telephone interviews were recorded in an Excel spread sheet. After that we performed validation to minimise data entry errors. For data analysis, we presented the information as a simple frequency distribution, using means and standard deviations (SD). We used the SAS package for Windows, version 9.2, 2002–2008 (Cary, NC, USA), for analysis.

## 3 Results

### 3.1 Impact on perinatal and maternal health

The number of deliveries in Roraima in 2019 before the pandemic was 13,811 and 2984 (21.6%) of these deliveries were of Venezuelan women and during the pandemic in year 2020 the total number of deliveries was 12,436 including 2300 (18.5%) deliveries of Venezuelan migrant women. This figure showed a decrease of 10% in the deliveries when compared 2019 with 2020 (Brasil, 2020c). This correlates with a decrease in the number of foetal deaths from 156 to 141 in the same period. However, the number of maternal deaths increased from 12 to 17 during the same period, with a maternal mortality ratio (for all women) of 86.9 and 136.7/100,000 births, respectively (Table 1). The number of deaths of pregnant women due to COVID-19 in Roraima state was nine (1.1%) out of the 880 in Brazil by July 2021, the second highest amongst the states of the northern region of Brazil (Brasil, 2021).

**Table 1:** Births, maternal and child mortality in Roraima state, Brazil (2019 and 2020).

Variables	Year 2019 n	Year 2020 n
Total number of births	13,811	12,436
Total number of births from Venezuelan women	2984	2300
Total number of maternal deaths	12	17
Maternal mortality ratio/100,000 live births	86.9	136.7
Total number of foetal deaths	156	141
Total number of neonatal deaths (<28 days)	151	167
Total number of deaths children under 1 year	150	84
Total number of deaths of children under 5 years old	374	288

### 3.2 Impact on health infrastructure

Healthcare managers reported that they needed to reallocate 223 (60.3%) out of the 370 beds available in the public network to treat COVID-19 cases. Furthermore, 14 of the 36 healthcare managers from the hospitals and health posts reported that at the state and municipal facilities some healthcare providers were reallocated to other units to assist in the pandemic healthcare. The providers did not report resistance to work in the assignments posed by the pandemic, they referred to a scarcity of healthcare providers and the resulting overload of tasks, difficulties of working using personal protective equipment (PPE), and a lack of information regarding the outpatient management of COVID-19 patients.

They also referred that the hospitals did not interrupt emergency services; but interrupted non-emergency surgeries and consultations. A total of 26 out of the 34 health posts assessed interrupted SRH services, including contraceptive provision and gynaecological consultation, since these services were not considered essential care. However, ANC consultations were offered on a regular basis.

### 3.4 Accessibility

Policy makers and managers of hospitals and health posts reported that digital technology/telehealth services were implemented to improve and facilitate the access to schedule appointments at the state and municipal levels, including for SRH care (WHO, 2018; Roraima, 2020). Most of the participants, (25/36; 69.4%) referred that the state and the municipal health authorities implemented community outreach using telephone, social media, radio, and television, with guidance for the population on how to schedule consultation using the healthcare network.

### 3.5 Challenges and opportunities for strengthening SRH services

All the professionals reported a proper medical waste and sharps disposal at all facilities. Regarding the availability of medical supplies for SRH care, the providers reported a lack of post-rape kits (76%), of contraceptive methods (67%), lack of supplies, medicines, and PPE (Table 2). In Table 3 we summarised the main problems reported by healthcare providers based on answers given to open-ended question. Each participant was allowed to give more than one answer. We grouped the answers with the same content even though reported in different ways. Many healthcare providers were afraid of infection with SARS-CoV-2, were concerned about the reduction of the number of providers, and had doubts about the management of the pandemic due to conflicting messages from the federal administration. According to 16 out of the 36 (44.4%) providers users of the public healthcare system had problems to reach the services due to lack or reduction of public transportation, or because they were afraid to be infected at the health facility.

**Table 2:** Assessment of the availability of some SRH medical supplies and disposal through the Covid-19 pandemic at hospitals (*n* = 3), women´s centres (*n* = 2) and health posts (*n* = 21).

Supplies and non-permanent materials availability	Yes n (%)
Kits for treatment after rape or violation	4 (15.4)
Stock of oral, injectable contraceptives and condoms	4 (15.4)
Stock of specific medicines for STI treatment	11 (42.3)
Stock of kits for IUD placement	7 (26.9)

IUD: intrauterine device; STI: sexual transmitted infections.

**Table 3:** Problems encountered and reported by healthcare providers and policy makers due to the COVID-19 pandemic (*N* = 39).

Problems encountered	n (%)
Healthcare providers afraid to acquire SARS-CoV-2	26 (72.2)
Reduction of number of healthcare providers	23 (56.5)
Relocation of providers to attend only COVID-19 patients	19 (41.3)
Conflicted messages received from the federal administration regarding the pandemic	18 (50.0)
Lack of equipment, supplies, and medicines including contraceptives	5 (13.9)

## 4 Discussion

Our study was initiated almost five years after the healthcare crisis imposed by the large number of Venezuelan migrants coming to Brazil who represented almost 10% of the population of the Roraima state. It is legitimate to speculate that as the healthcare system was mobilising existing resources to adapt to the strain posed by the increase number of people seeking healthcare, the advent of the COVID-19 pandemic overstretched the public healthcare system.

After the closure of the Brazil-Venezuela border due to the pandemic the flow of migrants reduced and the number of migrants reallocated from the UNHCR shelters to other parts of the country increased. This fact alleviated the demand for healthcare attention including the reduction of the number of deliveries (Brasil, 2020b; c). However, we do not have data concerning the number of ANC consultations and it is possible that many women did not attend all of the required ANC visits because they were concerned about the possibility of infection.

It is important to recall that the COVID-19 can potentially affects all persons, but many migrants and refugees are at heightened risk (OHCHR et al., 2020). Actions taken to deal with the emergency created by the COVID-19 has created imbalances in healthcare services. The needs for SRH care, the constraints of access to services and the culture barriers, from the perspective of Venezuelan migrant women in the region had been a source of concern (Albaladejo, 2018; UNHCR, 2019; Page et al., 2019; Doocy et al., 2019; Bahamondes et al., 2020; Asociación Profamilia and USAID, 2020; Makuch et al., 2021a; b; Soeiro et al., 2021a; b, Rivillas-García et al., 2021). These reports indicated that in Brazil when migrant women had access to services they reported to be satisfied with the attention care mainly obstetric care (Bahamondes et al., 2020; Makuch et al., 2021a). However, data from Colombia indicated that Venezuelan migrant women encounter barriers to access to SRH services (Asociación Profamilia and USAID, 2020; Rivillas-García et al., 2021). On the analysis of the data of our study we observed that services maintain ANC and other obstetric attention; however, not care in the other areas of SRH. It has been extensively described that migrant women, in general, need ANC, delivery and postnatal care; contraception provision; GBV care; and identification, treatment and guidance concerning STIs (Hussein, 2020; WHO, 2020b; Lau et al., 2020; USAID, 2021; Stirling Cameron et al., 2021).

Before the pandemic, Venezuelan migrant women at the same site reported that they were unable to obtain or have difficulties to obtain contraceptives, particularly LARCs, due to difficulties of access to services or lack of methods. One out of four women also reported difficulties to access ANC or postpartum care (Bahamondes et al., 2020). During the pandemic, the access of women aggravated due to the fact that 76% of the basic healthcare posts assessed interrupted the provision of SRH services, which are considered an essential services mainly in times of the COVID-19 pandemic (Bahamondes et al., 2020, World Health Organization; UNICEF; UNHCR 2012, World Health Organization 2020b), as reported also in Colombia, a country with large number of Venezuelan migrants (USAID, 2021). The WHO reported that one of the primary reasons for migrants not to seek medical attention were lack of availability of services, even though this reason is not exclusive for COVID-19 its may reflect every day challenges faced by migrants when they need health care (WHO, 2020c).

According to the information provided by the health authorities, many medical facilities were adapted to provide care for patients with COVID-19 and to allow patients to follow the recommended physical distance at the facilities. However, in many low- and middle-income public sector settings, the physical distance recommendations may be difficult to follow (WHO, 2020c). In these settings, medical appointments are usually not scheduled and patients experience long waiting times for health services in crowded waiting areas, a situation that can potentially increase the risk of virus transmission (WHO, 2020c; USAID, 2021). The physical distancing recommendations cannot be a barrier to offer SRH services, and new and innovative strategies must be implemented.

The healthcare providers reported re-allocation of professionals and many providers resigned due to fear of infection. This situation was also described in a survey from six countries that referred to the reallocation of healthcare providers from SRH care to the attention of COVID-19 cases (USAID, 2021). With the deployment of many healthcare providers and the increase in the number of COVID-19 cases, the cancellation of routine appointments became common. The pressure to obtain attention could be lower than before the COVID-19 because the number of potential users is lower and because users encounter problems related to access due to the reduction of public transportation (WHO, 2020c).

The strengths of our study was the broad view regarding the strains and challenges of healthcare services and providers due to the migrants crisis followed by the COVID-19 pandemic needs and the diversity of our sample, which included policy makers, managers, and providers. We can consider a possible limitation the fact that we focus on service functioning and organisation rather than service uptake from the providers involved. Further, we cannot affirm that the problems encountered in the provision of SRH services to Venezuelan migrant women during the pandemic were not encountered in the provision of services to local national women. Nevertheless, the objective of our study was to assess the possibilities to offer SRH services for Venezuelan migrant women. We presume that the difficulties of access and constraints to offer these services are similar for all women. In our results, we did not find information on specific restriction of service provision to migrant women.

Our results showed several gaps in the current health system, including problems with infrastructure, and lack of commodities. During the last two years, healthcare attention has been concentrated on COVID-19 cases, leading, amongst other essential health issues, to the neglect of SRH care, which is an important healthcare issue for women (Hussein, 2020; Stirling Cameron et al., 2021). Access to contraceptive counselling and provision, ANC visits, safe assisted deliveries, postnatal care amongst others are essential forms of women healthcare, and any reduction or barriers posed to these services is concerning (WHO, 2020c; USAID, 2021). Under the pandemic there was a reduction in SRH services, consequently it is legitimate to suppose that women suffered constraints and encounter difficulties in their access to these services and has impacted SRH rights with consequences that need to be study in the future.

One of the principles of the WHO and the Brazilian NHS is universal health coverage (Campos, 2018, World Health Organization 2019), but the system must be properly prepared to ensure access for all people at all times to healthcare services when needed. There is a need of cooperation of all the administrative levels within the healthcare network to pull together all the lessons learn along the pandemic to implement effective interventions to deal with the pandemic and continue offering essential health services to women (Lancet, Editorial, 2020; WHO, 2020a, 2020b, 2020c).

## 5 Conclusion

The healthcare system in Roraima was impacted firstly by the need to provide SRH to a large number of Venezuelan migrant women, and after an effort to adapt to the reality that this migrant crisis posed; this system was affected by the needs to adapt to the COVID-19 pandemic. Due to the scare information about how services prepare to offer SRH during the pandemic, our findings may be a relevant contribution for policy makers, stakeholders and managers, in order to elaborate strategies to improve access to care and quality of care in SRH during pandemic time.

## Funding

This work was funded by the CEMICAMP, center for Research in Reproductive Health of Campinas.

## Authorship contribution statement

LB, MYM designed the study. LB performed the first visit to established all the contacts before to start the data collection. CB and HSF de A facilitated the data collection and participated in the analysis. LB, MYM performed the data collection. LB, MYM contributed with the analysis and LB, MYM wrote the first draft. All the authors interpreted the data and co-wrote the manuscript/substantive editing and review and approved the final manuscript.

## Declaration of Competing Interest

The authors have declared that no competing interests exist.
